# Oxidative Stress Markers in Sputum

**DOI:** 10.1155/2016/2930434

**Published:** 2016-01-14

**Authors:** Balazs Antus

**Affiliations:** ^1^Department of Pathophysiology, National Koranyi Institute of TB and Pulmonology, Piheno Utca 1, Budapest 1121, Hungary; ^2^Department of Pulmonology, National Koranyi Institute of TB and Pulmonology, Piheno Utca 1, Budapest 1121, Hungary

## Abstract

Although oxidative stress is thought to play a pivotal role in the pathogenesis of inflammatory airway diseases, its assessment in clinical practice remains elusive. In recent years, it has been conceptualized that oxidative stress markers in sputum should be employed to monitor oxidative processes in patients with asthma, chronic obstructive pulmonary disease (COPD), or cystic fibrosis (CF). In this review, the use of sputum-based oxidative markers was explored and potential clinical applications were considered. Among lipid peroxidation-derived products, 8-isoprostane and malondialdehyde have been the most frequently investigated, while nitrosothiols and nitrotyrosine may serve as markers of nitrosative stress. Several studies have showed higher levels of these products in patients with asthma, COPD, or CF compared to healthy subjects. Marker concentrations could be further increased during exacerbations and decreased along with recovery of these diseases. Measurement of oxidized guanine species and antioxidant enzymes in the sputum could be other approaches for assessing oxidative stress in pulmonary patients. Collectively, even though there are promising findings in this field, further clinical studies using more established detection techniques are needed to clearly show the benefit of these measurements in the follow-up of patients with inflammatory airway diseases.

## 1. Introduction

Oxidative stress plays a pivotal role in the development of many lung diseases associated with chronic airway inflammation such as asthma [1, 2], chronic obstructive pulmonary disease (COPD) [3, 4], and cystic fibrosis (CF) [5]. Increased oxidative stress in these conditions derives from the burden of inhaled oxidants and reactive oxygen species (ROS) generated by several inflammatory and structural cells of the airways. The increased production of ROS raises oxidative lipid peroxidation and protein/DNA damage and is thought to aggravate airway inflammation via multiple mechanisms including proinflammatory mediators and effects on smooth muscle and mucus secretion. On the other hand, damaged antioxidative defense mechanisms, altered homeostasis of the airway surface liquid (ASL), and acute or chronic airway infections (bacterial colonization) could also contribute to enhanced oxidative stress in these diseases [6, 7].

Although oxidative stress has been studied in inflammatory airway diseases for decades, its reliable assessment in clinical practice has remained elusive. A number of local (lung-specific) [8, 9] and systemic (blood-based) [10, 11] oxidative stress markers have been suggested to serve as indicators of oxidant-induced tissue damage in the lungs. While elevated levels of putative markers in plasma indicate systemic oxidative stress that may or may not originate in the respiratory tract, assessment of markers in respiratory samples is more likely to reflect oxidative processes that occur in the lungs.

Among the different techniques of sampling the respiratory tract, bronchoscopy and bronchoalveolar lavage (BAL) are invasive procedures [8, 12]. They may cause discomfort to the patients and may not be possible to apply to patients with more severe disease and repeated measurements are difficult to perform. In contrast, exhaled breath condensate (EBC) and sputum collections are noninvasive and semi-invasive procedures and, thus, they are more likely to gain a foothold in clinical practice [8, 9, 12, 13]. These techniques are safe, do not require special invasive intervention, and can be repeated within a relatively short period of time. These methods offer a unique opportunity to identify pulmonary biomarkers of potential clinical utility in the management of airway diseases.

In recent years, several markers and “footprints” of oxidative/nitrosative damage have been detected both in EBC and in sputum [8, 9, 12, 14]. However, measurement of putative mediators in EBC has usually poor reproducibility and marker concentrations are often very close to the detection limit of the assay [15, 16]. Moreover, biomarkers may be affected by the sampling procedure itself, as acknowledged also in the European Respiratory Society (ERS) Task Force Report [17]. For example, there is evidence that in EBC the concentration of hydrogen peroxide (H_2_O_2_), a well-known marker of oxidative stress, depends on both the expiratory flow rate [18] and the breathing pattern during sample collection [19]. Finally, the variable dilution of ASL droplets by water vapor has also been recognized for many years as an important confounding factor in EBC assays [17]. The water vapor is generated as a gas in the lungs and only becomes a liquid with cooling. In order to assess the dilution of EBC samples by water vapor in different conditions, different candidate markers (urea, total cations, and conductivity) have been introduced. However, the accurate measurement of these indicators in EBC is problematic. Moreover, their potential usefulness is based on the assumption that concentrations of each indicator in the ASL are similar in the plasma which again may be incorrect. Thus, as it stands now, due to the several technical and methodological limitations of EBC assays, the method is not likely to be used in clinical practice in the near future.

In contrast, assessment of oxidative stress markers in sputum may be a more reliable approach to study the relationship between airway inflammation and oxidative tissue injury. Sputum can be considered as a bio-gel with a high concentration of different types of cells and enzymes. It is less diluted with water than EBC and, thus, the levels of putative markers are 5- to 10-fold higher in the sputum than in the EBC that allows more accurate detection [20]. Assessment of sputum cell profile provides direct information on the inflammatory cells present in the airways. Sputum is usually obtained by induction using hypertonic saline, which is a standardized procedure, and recommendations for sputum induction have been formulated by the ERS/American Thoracic Society (ATS) Task Force [21]. Since it is generally believed that sputum accurately mirrors conditions at the site of oxidative damage in the airways [8, 9, 12, 22], measurement of oxidative stress products in sputum may have clinical relevance. Sputum samples, however, need to be processed within a relatively short period of time, and the evaluation of the cell fraction requires expertise. Thus, sputum analysis cannot be performed in primary care settings without laboratory background.

In this review, oxidative stress-derived products in the sputum were briefly discussed: in order, first, to demonstrate the current body of evidence supporting their application as biomarkers in the management of inflammatory airways diseases and, second, to identify gaps in knowledge which should be further investigated in the future.

## 2. Aldehydes

**Figure 1:**
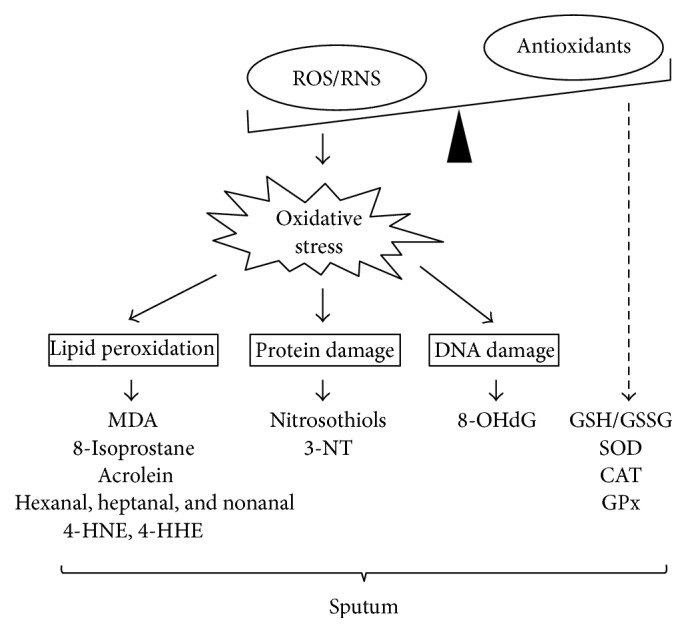
Different forms of oxidative stress-related tissue injury and their potential, sputum-based biomarkers. ROS: reactive oxygen species, RNS: reactive nitrogen species, MDA: malondialdehyde, 4-HHE: 4-hydroxyhexanal, 4-HNE: 4-hydroxynonenal, 3-NT: 3-nitrotyrosine, 8-OHdG: 8-hydroxy-2′-deoxyguanosine, SOD: superoxide dismutase, CAT: catalase, GSH: glutathione, GSSG: glutathione disulfide, and GPx: glutathione peroxidase.

Among the different forms of oxidative stress-induced tissue injury, lipid peroxidation has been the most extensively investigated [23]. During this process, a number of different lipid hydroperoxides and aldehydic products are formed; from those, malondialdehyde (MDA) has been the most frequently studied as a marker of oxidative stress in various pulmonary diseases (Figure 1). MDA not only is a marker for oxidative decomposition of polyunsaturated fatty acids (PUFAs) but also might have important atherogenic, mutagenic, and cancerogenic actions, as it is capable of forming different, biologically relevant DNA and protein adducts [24, 25].

A number of different methods have been developed for measuring MDA in a variety of different matrices including sputum supernatant [26]. In general, analytic methods can be subdivided into derivatization-based and label-free methodologies. These strategies have been further coupled to separation techniques such as liquid chromatography (LC) and gas chromatography (GC). Among label-free techniques, the simple ultraviolet (UV) absorbance-based method has poor sensitivity and specificity. Other separation techniques such as reverse-phased LC [27], capillary electrophoresis (CE) [28], or LC-tandem mass spectrometry (MS/MS) [29] are more advanced and accurate techniques that can be applied for both EBC and sputum.

The most frequently applied derivatization-based method is the thiobarbituric acid (TBA) assay, in which condensation of two molecules of TBA with one molecule of MDA gives a colored reaction product [26]. This compound can then be easily measured spectrophotometrically or by fluorescence detection. Nevertheless, TBA assay is not specific for MDA, and in complex biological systems including the sputum many compounds (simple and complex carbohydrates, protein, and nucleic acid oxidation products) can react with TBA to produce colored adducts [30]. Thus, one cannot directly equate the measurement of TBA-reactive substances with MDA or the degree of lipid peroxidation in the airways. The assay should be combined, for example, with LC separation and fluorescence detection of the formed product [31].

Other novel approaches for the specific determination of MDA include hydralazine-based derivatization methods that are again coupled with high-performance liquid chromatography (HPLC) or LC/atmospheric pressure chemical ionization tandem mass spectrometry (LC/APCI-MS/MS) [32, 33]. These techniques are very sensitive and reliable. For example, when the measurement is performed by HPLC, after samples preparation, the resulting fluorophore is a highly specific product, which is detectable at very low levels using a fluorescence detector. Nonetheless, applicability of these assays to large sample cohorts might be difficult due to the complexity of the methods. Finally, several hydralazine- and non-hydralazine-based derivatization procedures have also been described and used for the analysis of MDA by GC-MS/MS [26].

Using the LC-MS/MS methodology, Corradi et al. have reported for the first time that MDA levels in induced sputum are elevated in stable asthma and COPD patients compared to healthy controls [34]. Additionally, our research group has recently demonstrated that MDA concentrations in sputum, but not in EBC, are further increased in COPD exacerbations [35]. Treatment of exacerbation with bronchodilators and systemic corticosteroids led to a decrease in sputum MDA levels, primarily in those patients who had more pronounced improvement in airflow limitation after treatment. Measurement had good reproducibility; coefficients of variation for intra- and interassay repeatability were 6.6 and 9.1%, respectively. Of importance, MDA levels did not correlate with lung functional parameters indicating that airflow limitation by itself does not determine the degree of lipid peroxidation in the airways. Inflammatory cell counts in the sputum were not related to MDA values either.

As demonstrated recently in another study from our laboratory, sputum MDA concentrations are markedly increased in CF patients as well [36]. In fact, measurement of MDA in sputum discriminated between patients and controls with greater accuracy than in plasma, where MDA levels were also increased but to lesser extent. We speculated that, in the blood, the detection of imbalance between oxidant and antioxidant statuses might be more difficult due to the number of systemic confounding factors (comorbidities, nutrition, etc.). Like in COPD, MDA and spirometric values did not directly correlate in CF patients. Nonetheless, patients with more impaired pulmonary function (forced expiratory volume in 1 sec [FEV_1_] < 50%) had significantly elevated concentrations of MDA in sputum compared to those with mild-to-moderate pulmonary dysfunction (FEV_1_ > 50%) indicating that there is yet some relationship between the level of oxidative stress and the degree of lung tissue damage. Of importance, significant difference was detected only using respiratory samples. Thus, again, measurement of MDA in sputum but not plasma may be useful for assessing oxidative stress in CF.

Besides MDA, oxidation of the cell membrane phospholipids results in the formation of various other aldehydic products including hexanal, heptanal, nonanal, acrolein, 4-hydroxyhexanal (4-HHE), and 4-hydroxynonenal (4-HNE) (Figure 1). While *α*,*β*-unsaturated aldehydes (4-HHE, 4-HNE) are generated mainly by the peroxidation of *ω*-6 (e.g., AA and linoleic acid) and *ω*-3 (e.g., oleic acid) PUFAs, saturated aldehydes (hexanal, heptanal, and nonanal) are known to be breakdown products of oxidized linoleic, arachidonic, palmitoleic, and oleic acids [37].

Most of these compounds appear to be detectable in the sputum supernatant of patients with asthma and COPD using the LC-MS/MS methodology. In the study of Corradi et al. [34], it has been found that acrolein and hexanal levels were significantly increased in patients with asthma and COPD compared to healthy subjects, while concentrations of 4-HHE and 4-HNE were similar between patients and controls. Nonanal levels were increased only in patients with COPD but not asthma. Aldehyde levels showed no correlation with sputum differential cell counts or lung function variables. The intra-assay variability of aldehyde measurements in this study was within 2–8% for all products.

Some limitations of these measurements deserve comments. First, there is evidence that cigarette smoking alone increases levels of MDA and other saturated aldehydes in the airways [33]. Thus, smoker and nonsmoker pulmonary patients should be separately investigated, which could be difficult in clinical settings. Unfortunately, in some trials [34], the group with COPD included current smokers as well, which might have affected the overall outcome of the study. Second, most of the studies were cross-sectional or, when longitudinal, followed up patients for only short periods of time. Temporal variations in marker levels might occur irrespective of the pulmonary status of the patients, which may limit the clinical applicability of the test. Third, sputum concentrations of some markers such as 4-HHE and 4-HNE lay very close to the detection limits of the assay which makes data interpretation difficult. Finally, the source of aldehydes in sputum remained elusive, and it is not clear whether inflammatory or epithelial cell membrane lipids were primary affected by lipid peroxidation in these disorders.

## 3. 8-Isoprostane

Isoprostanes represent a unique group of arachidonic acid (AA) derivatives, since they are produced nonenzymatically from AA during the peroxidation of membrane lipids and, in addition to having relevant biological activities, may also be potential useful markers of oxidative stress [38]. Isoprostanes appear in various body fluids including the plasma and the urine under normal conditions and are elevated by oxidative stress.

Among these AA derivatives, 8-isoprostane, also known as 8-epi-prostaglandin F_2*α*_ (8-epi-PGF_2*α*_), is one of the most commonly investigated lipid peroxidation markers in pulmonary diseases (Figure 1). It is a potent pulmonary and renal vasoconstrictor [39] and has been implicated as a causative mediator of pulmonary oxygen toxicity [40]. 8-Isoprostane can be detected easily in various respiratory samples including sputum by commercially available enzyme immunoassays (EIA). However, measurement has often considerable variability. The combination of GC and/or LC-MS methodology offers increased sensitivity for analysis, but these techniques are more expensive and time consuming than EIA. Besides 8-isoprostane, there are a number of other stereoisomers of PGF_2*α*_ that might have biological activities and could also serve as markers of lipid peroxidation in different conditions.

Several studies showed higher 8-isoprostane levels in the sputum of patients with stable asthma [41], COPD [42, 43], or bronchiectasis [41] compared to healthy controls. However, in mild asthmatics, sputum 8-isoprostane levels appear to be normal [44], and there are reports where no change in 8-isoprostane values was observed even in severe asthmatics [45]. The reasons for these discrepancies are not clear; however, methodological factors such as variances in the sensitivity of EIA kits or effects of sample storage may contribute to different study outcomes.

There is evidence that sputum 8-isoprostane concentrations are further elevated during acute asthma and decrease along with treatment/recovery [41]. Similarly, in COPD exacerbations, sputum 8-isoprostane levels are further increased, as documented recently by our laboratory [46]. These results are in agreement with the general view that both asthma and COPD exacerbations are accompanied by enhanced inflammation and ROS generation in the airways. Nonetheless, our data also indicate that a successful hospital treatment resulting in clinical and functional recovery of the patients does not completely abolish the increased oxidative stress observed in exacerbation by the time of patient's discharge from the hospital. Delayed resolution of inflammatory response during recovery from an exacerbation may be responsible for this phenomenon [47]. Alternatively, variances in treatment regimens during hospitalizations could play a role, since treatment was not standardized in this trial [46].

Few studies have investigated 8-isoprostane in sputum of CF patients. Interestingly, marker levels were elevated only in acute but not stable CF patients [48]. Antibiotic treatment in acute patients did not affect 8-isoprostane concentrations, although clinical improvement of the patients has been observed. It can be speculated that high proportion of dead or lysed cells and their debris represent the major source of 8-isoprostane in sputum supernatant in this condition, and therefore, 8-isoprostane may be less suitable for use as a marker of oxidative stress in CF.

The relationship between lung function (FEV_1_) and sputum 8-isoprostane level is inconsistent. In some studies, a significant negative correlation was documented [42, 48], while in other series no association was found [46]. Investigating the inflammatory cell profile of the sputum, the number of neutrophils is usually related to 8-isoprostane levels [42, 46]. Interestingly, in our study, the lymphocyte cell counts showed also a strong association with 8-isoprostane values in the sputum [46].

Cigarette smoking appears to be an important confounding factor in 8-isoprostane measurement. There is evidence that smoking alone significantly increases levels of sputum 8-isoprostane when compared to nonsmoking controls [42, 43]. In some studies, even the pack-year index was significantly related to 8-isoprostane concentrations [42]. Effect of smoking is rather long-lasting, as Louhelainen et al. have documented that 8-isoprostane levels remain elevated even three months after patients stop smoking indicating ongoing oxidative stress in the lungs [49].

Although these data suggest that 8-isoprostane might be a useful airway marker of oxidative stress in some diseases, several questions should be addressed in further studies. First, since 8-isoprostane levels are highly variable, especially in asthmatics [41, 44], more studies assessing day-to-day and between-visit variability of the measurement are needed. Furthermore, interventional studies are needed to investigate the effects of corticosteroids (inhaled or oral), *β*_2_-agonists, and antimuscarinic drugs on 8-isoprostane levels in the sputum during the follow-up of patients with inflammatory airway diseases. Even though these issues will be adequately addressed in the future, the confounding effect of smoking on 8-isoprostane measurements remains a major limitation, particularly in COPD patients. It is a common experience that even those patients, who are hospitalized due to an acute exacerbation of the disease, often continue to smoke during treatment, and the compliance of smokers with hospital no-smoking policies is generally poor. Therefore, measurement of 8-isoprostane may gain a foothold in clinical practice only in the management of ex-smoker or nonsmoker subjects.

Finally, it can be noted that dithiothreitol (DTT), which is a small-molecule redox agent used commonly for sputum homogenization, may have a confounding effect on 8-isoprostane EIA measurement. To circumvent this effect, it is recommended that DTT of the same concentration as in the sputum supernatant should be added to the standards (standard curve), when performing the assay [46, 50]. Of importance, not all markers are affected by DTT. For example, data from our laboratory indicate that measurement of MDA was not influenced by the presence of DTT in the sputum [35]. Nonetheless, MDA was determined by HPLC and not EIA. Thus, it is reasonable that the effect of DTT should be tested separately for each marker in the sputum.

## 4. Nitrosothiols

Reactive nitrogen species (RNS) are a diverse group of nitric oxide- (NO^∙^-) derived oxidants that act together with ROS to damage cells, causing nitrosative stress (Figure 1) [51]. In contrast to nitrous oxide (N_2_O), NO^∙^ contains odd number of electrons and is therefore a highly reactive free radical that is stabilized ultimately as nitrite (NO_2_^−^) and nitrate (NO_3_^−^) or in biological complexes with thiols to generate nitrosothiols.

A series of reports over recent years have demonstrated signs of increased nitrosative stress in asthma and COPD [51, 52]. Nitrosative stress has been often linked with the excessive NO^∙^ production by the inducible type of NO synthases (iNOS, NOS2). In contrast, NO derived from the constitutive type of NOS (cNOS, NOS1, and NOS3) is thought to induce bronchodilation and pulmonary vasodilatation to maintain homeostasis in normal conditions. There is good evidence indicating that enhanced RNS formation promotes airway inflammation and airway hyperresponsiveness in asthma [53], while in COPD RNS have been suggested to particularly contribute to activation of matrix metalloproteinase and inactivation of antiproteases [54]. However, increased formation of RNS is also part of the unspecific defense system of an organism against, for example, bacteria and other microbes [55, 56].

Nitrosothiols can be quantified by commercial available assays in different body fluids. The most popular and simple method for the determination of nitrosothiols is the* Saville* reaction involving the treatment of nitrosothiols with mercuric chloride, which releases NO_2_^−^ that then reacts with* Griess* reagents to form an azo dye that can be detected colorimetrically. Other techniques such as chemiluminescence-based methods are more sensitive and accurate for the detection of nitrosothiols in biological fluids [57]. Nitrosothiols are thought to play an important role in the regulation of vasodilatation, platelet aggregation, and leukocyte adhesion in various pathological conditions [58].

So far, only few studies have investigated nitrosothiols in the sputum of pulmonary patients. There is some evidence that nitrosothiols are increased in patients with COPD compared to healthy controls and that levels of nitrosothiols correlate with the number of neutrophils in the sputum [59]. Increased nitrosothiol levels have been reported in eosinophilic bronchitis as well [60]. Nonetheless, the clinical relevance of these findings remained poorly understood. More studies are needed in order to properly define both the variability and the reproducibility of these measurements and the potential of this marker as a clinical tool in monitoring disease activity, for example, the course of exacerbation of inflammatory airway diseases.

The relationship between nitrosative stress and levels of fractional exhaled nitric oxide (FENO) in pulmonary patients is uncertain. While increased iNOS expression in the airways is thought to be the main cause of higher FENO levels in patients with asthma, most studies report similar or only slightly increased FENO levels in COPD patients despite increased nitrosative stress in these patients [8, 11–14]. FENO levels do not appear to directly correlate with sputum nitrosothiol levels in COPD patients either [59].

## 5. Nitrotyrosine

It is well known that the reaction of NO^∙^ and superoxide anion (O_2_^∙−^) leads to the formation of peroxynitrite (ONOO^−^), which in turn exerts various harmful effects in the airways [52]. On one hand, ONOO^−^ is a powerful oxidant, which is able to enhance the formation of other even more reactive free radicals including the hydroxyl radical (OH^∙^) in cellular milieu. Moreover, ONOO^−^ can react with a wide variety of molecular compounds including DNA, lipids, and sulphydryl group of proteins to promote nitrosative stress. Similarly, ONOO^−^ reacts with the tyrosine residues of proteins to form a stable product such as 3-nitrotyrosine (3-NT). Nitration of tyrosine residues appears to inactivate numerous enzymes and prevent kinase substrate phosphorylation, suggesting that tyrosine nitration not only gives rise to inactive “footprints” of nitrosative stress but may also have a functional relationship with the pathophysiology of inflammatory airway diseases [61]. In line with this view, it has been proposed that 3-NT plays a major role in the development of airway remodeling [62] and it contributes to airway hyperresponsiveness and epithelial damage in asthma [63].

In most studies, 3-NT is measured by EIA kits. Again, however, there are other more reliable analytic techniques (GC-MS/MS, LC-MS/MS, or HPLC) also available [64, 65].

Investigating the degree of protein nitration in pulmonary patients, it has been shown that sputum inflammatory cells exhibit marked 3-NT immunoreactivity in subjects with COPD and to a lesser extent in those with asthma, but not in healthy controls [66]. In patients with COPD but not asthma, the amount of 3-NT formation shows a significant negative correlation with FEV_1_ values. However, sputum samples of smokers without airway obstruction and also some samples of nonsmokers may display increased number of 3-NT positive cells as well [67]. Thus, based on the number of 3-NT positive inflammatory cells in the sputum, the degree of nitrosative stress in the airways cannot be adequately estimated in COPD or asthmatic patients.

However, there is also evidence that in COPD exacerbations the number of 3-NT positive macrophages and polymorphonuclear cells in the sputum is further increased suggesting that these subjects have more nitrosative stress in the airways [68]. Similarly, 3-NT levels are elevated in patients with refractory asthma compared to the well-controlled group [69]. This implies that measurement of 3-NT may assist in the selection of patients who would potentially respond worse to treatment. In eosinophilic and noneosinophilic asthma, however, the number of 3-NT positive sputum cells seems to be similar [70] indicating that eosinophils may not be directly involved in the development of nitrosative stress in asthmatic subjects.

Signs of increased nitrosative stress can be detected in CF as well. In keeping with this notion, Jones et al. documented recently that NO_3_^−^ and 3-NT levels, but not NO_2_^−^, were significantly elevated in the sputum of patients with CF [71]. Concentration of 3-NT showed a significant correlation with the level of myeloperoxidase (MPO), an enzyme that is commonly recognized as a marker of neutrophil activity and has also been implicated in the mechanism of NT formation in the airways. Although high levels of active MPO and elevated amounts of MPO characteristic protein oxidation products were observed in other studies as well, it appears to be that the degree of protein nitration does not necessarily correlate with indexes of epithelial toxicity in CF [72]. In agreement with these data, our recent study indicated that the extent of lipid peroxidation, as assessed by the level of MDA in the airways, and the sputum concentration of neutrophil elastase (NE), another common marker of neutrophil activations and a major effector of tissue damage in CF, were not directly related in CF patients either [36].

Finally, there is some evidence that 3-NT levels can be modified upon treatment with bronchodilators or anti-inflammatory agents. For example, it has been shown that long-acting *β*_2_-agonists and antimuscarinic drugs modulate iNOS protein expression and 3-NT levels via the signal transducer and activator of transcription-1 (STAT-1) pathway in human bronchial epithelial cell lines [73]. Moreover, Hirano and colleagues have documented that treatment with both theophylline and inhaled corticosteroids (ICS) reduces the number of 3-NT positive sputum cells as well as the amount of 3-NT in sputum supernatant in patients with COPD [74]. Drugs were administered for 4 weeks in a randomized crossover manner with a washout period of 4 weeks. Interestingly, the effect of theophylline was more pronounced. In line with this finding, Sugiura et al. have also demonstrated that a 4-week treatment with ICS reduced both the NT and the iNOS immunoreactivity in sputum cells of COPD patients when compared with pretreatment levels [75]. The reduction in NT and iNOS immunoreactivity correlated with improvement in FEV_1_ indicating a direct relationship between the degree of nitrosative stress and lung function. This finding might be relevant primarily for those COPD patients who have more eosinophils in their sputum and thus exhibit increased corticosteroid responsiveness. Unfortunately, most patients have neutrophilic but not eosinophilic airway inflammation and respond poorly to ICS regimens.

## 6. Markers of DNA/RNA Damage

Oxidative stress induces not only lipid peroxidation but also the damage of other cellular macromolecules such as nucleic acids. Thus, oxidative modifications of these molecules may also serve as markers of oxidative injury (Figure 1).

Oxidative damage to DNA/RNA may be particularly important in COPD [76]. Guanine is the base most prone to oxidation that leads to the formation of 8-hydroxy-2′-deoxyguanosine (8-OHdG) from DNA, 8-hydroxyguanosine from RNA, and 8-hydroxyguanine from either DNA or RNA. It has been proposed that, in susceptible subjects, cigarette smoke injures the airway epithelium generating the release of endogenous intracellular molecules, whose signals are then captured by antigen presenting cells and are transferred to the lymphoid tissue, generating adaptive immune response and enhanced inflammation and oxidative stress [77]. The insufficiency of DNA repair is also a common finding in COPD [78, 79]. Moreover, there is evidence that the frequent exacerbator phenotype exhibits the highest rates of genetic defects indicating that increased inflammation and oxidative burden on cells during exacerbations might lead to greater oxidative DNA damage [80]. This is an important point since other studies have also revealed that persistent systemic inflammation is associated with poor clinical outcome, and COPD patients with elevated inflammatory marker levels in blood belong to a distinct clinical phenotype [11]. Thus, it can be speculated that markers of DNA/RNA damage may assist in the selection of high-risk patients.

Nonetheless, only few studies have investigated markers of DNA/RNA damage in the sputum so far. Investigating stable COPD patients, Tzortzaki et al. found that 8-OHdG levels were significantly increased in these subjects when compared with patients with bronchiectasis, smokers without COPD, or healthy controls [81]. Proklou et al. evaluated asthmatics with different smoking habits and showed that the highest 8-OHdG levels could be detected in smoking asthmatics [82]. Levels were slightly reduced in nonsmoking asthmatics, while the lowest levels were found in healthy, nonsmoking subjects. In all these studies, measurement of 8-OHdG was performed by enzyme-linked immunosorbent assay (ELISA). Unfortunately, no data on repeatability and/or variability of the measurements were reported.

## 7. Antioxidants

It is well known that antioxidant enzymes such as superoxide dismutase (SOD), catalase (CAT), or glutathione (GSH) defend against deleterious consequences of a wide variety of ROS and RNS produced endogenously in the lungs and sometimes accessed through exposure to the environment (Figure 1). Impaired antioxidant defenses have been described in many respiratory diseases including asthma, COPD, and CF [1–6].

Among these enzymes, GSH has been implicated as the major antioxidant of the human airway secretion [8]. GSH is a tripeptide with many physiological functions. Protecting the cells against ROS, GSH can act in two ways: first, GSH reacts directly with free radicals (O_2_^∙−^, OH^∙^, etc.) in nonenzymatic reactions; and second, it acts as an electron donor in the reduction of peroxides catalyzed by glutathione peroxidase (GPx). In this reaction, glutathione disulfide (GSSG) is formed. GSSG can be reduced again to GSH by the action of the enzyme glutathione reductase. In general, the ratio of GSH/GSSG can be used as a marker for oxidative stress within the cells. Of importance, under normal conditions, more than 90% of blood GSH is found as free GSH. A change of the equilibrium towards oxidized GSH (GSSG) points to an increased generation of ROS thus being an indicator of increased oxidative stress. GSH is usually determined in the blood; however, measurement in the sputum is also feasible and has good repeatability [83].

In theory, attenuation or loss of antioxidant activity may indicate increased oxidant-induced tissue injury. However, studies provided conflicting results. For example, Dauletbaev et al. have demonstrated that total GSH levels are increased in patients with CF compared to non-CF individuals [84]. Likewise, Beeh et al. have shown that both total and oxidized GSH are increased in the sputum of patients with moderate-to-severe COPD and that levels of oxidized GSH were positively correlated with sputum neutrophils [59]. Increased sputum concentrations of CAT have also been documented in patients with CF [85]. Furthermore, Dauletbaev and coworkers showed that CF sputum was capable of preventing intracellular oxidant accumulation in cells incubated with high doses of H_2_O_2_ in an* in vitro* cytotoxicity model indicating that sputum from these subjects may have profound antioxidant properties [86].

By contrast, Zeng et al. have reported that SOD, GSH, and GPx levels in the sputum were lower in stable COPD patients compared to healthy smoker and nonsmoker subjects and that these enzyme levels further decrease during acute exacerbations [87]. With regard to activity of SOD in COPD exacerbations, just the opposite was found in a pilot study from our laboratory [88]. The discrepancies between the studies may be due to the differences in study populations: in our study [88], severe, hospitalization-requiring exacerbations were evaluated and all subjects were ex-smokers, while in the trial of Zeng et al. [87] only milder cases (patients without systemic corticosteroid treatment) were taken into account and both smokers and ex-smokers were included. Based on data reported in the literature, systemic antioxidant levels show also great heterogeneity in patients with CF or COPD [89, 90].

In patients with asthma, studies report elevated [91, 92] or normal [93] GSH levels. Additionally, a recent trial indicated that the glutaredoxin- (GRX-) 1/S-glutathionylation (PSSG) axis is also altered in asthmatics [94]. As discussed elsewhere [95], GRXs are involved in the regulation of the GSH redox cycle and participate both in GSH binding to proteins and in oxidation/reduction of thiol containing enzymes, while PSSG protects targeted thiols from irreversible oxidations and can modulate protein function. In patients with COPD, GRX-1 is mainly expressed by alveolar macrophages and the number of GRX-1 positive macrophages negatively correlates with FEV_1_ [96]. In the same study, authors have also documented that sputum GRX-1 protein expression was higher during the course of COPD exacerbations suggesting a relationship between disease activity and alterations in GSH homeostasis. However, in lung homogenates, GRX-1 expression was reduced in COPD patients compared to smokers and nonsmokers without COPD. Again, diverse study outcomes may be explained on numerous confounding factors (smoking status, comorbidities, nutritional intake of antioxidants, etc.) affecting the antioxidant status of the patients. Hence, as it stands now, assessment of oxidative stress on the level of antioxidants in the sputum appears to be difficult, and further studies are needed to evaluate the relevance of these measurements in the management of pulmonary patients.

## 8. Conclusions and Future Challenges

Oxidative stress defines an imbalance between formation of ROS and antioxidative defense mechanisms. Both increased production of ROS and attenuation of antioxidant activity can induce oxidative stress. The balance, however, is rather fragile, difficult to predict, and strongly dependent on environmental conditions.

Several recent studies have used sputum as a type of respiratory sample to study oxidative stress in inflammatory airway diseases. As mentioned above, sputum is usually obtained by induction with hypertonic saline, although some patients, especially those with chronic bronchitis or CF, have often spontaneous sputum as well. Sputum induction is a semi-invasive process that is safe, effective, and relatively reproducible and is feasible to perform even in patients with acute exacerbations [97, 98]. Although induction can induce a low-grade inflammatory response in the airways, the process by itself has no effect on 8-isoprostane or MDA readings in the sputum [35, 46]. The most important limitation of sputum analysis is the fact that both processing and evaluation require expertise. Furthermore, appropriate laboratory background is also necessary, since processing should be performed within 2 hours in general.

According to the published data, a number of different oxidative stress markers can be detected in the sputum of patients with asthma, COPD, or CF. Among these molecules, 8-isoprostane, MDA, and 3-NT appear to be the most promising markers, but even their measurements are often performed by nonreliable techniques, which must be taken into account when interpreting the findings. The most commonly detected oxidative stress markers in sputum are summarized in Table 1.

One of the most important limitations of these measurements is high variability. The reason for high marker variability in sputum is likely to be multifactorial and may be explained by changes in the composition of the ASL, variations in the rate of contamination of the sputum samples with saliva, or differences in the measurement techniques.

In the light of these concerns, first, studies should employ more established and standardized detection techniques in order to gain more reliable and reproducible results. For example, as emphasized recently by Forman et al. [30], the use of TBA assay in a sole indicator of lipid peroxidation in a complex biological system such as sputum is not appropriate. Similarly, antibodies used in most 8-isoprostane ELISA/EIA kits are not specific enough. It can be recommended that TBA products, particularly MDA, and F2-isoprostanes in sputum should be measured by MS to obtain adequate estimates of lipid peroxidation.

Second, interventional studies are needed in order to assess the effect of treatment with ICS and short- or long-acting bronchodilators on oxidative markers. It would be reasonable to incorporate these biomarker measurements into large-scale clinical trials that are performed in specialized centers capable of sputum induction and processing. Furthermore, it should be emphasized that both asthma and COPD contain many phenotypes, and in the various phenotypes different patterns of inflammatory cells and mediators are involved. Studies should explore the relationships between oxidative marker patterns and distinct clinical phenotypes.

Finally, the prognostic value of these markers should be evaluated in terms of long-term disease outcomes. Studies should explore whether the exacerbation frequency and the rate of functional decline in asthma or COPD patients vary according to the degree of oxidant/antioxidant imbalance in the airways. In patients with COPD or CF, chronic bacterial infections of the airways are common and may also contribute to the dysregulation of redox balance in the lungs, which should be taken into account. Thus, a major task for the coming years will be to evaluate oxidative stress markers in sputum more rigorously and to analyze them in clinical interventional studies with hard end-points to prove their usefulness in clinical practice.

## Figures and Tables

**Table 1:** Oxidative stress markers in sputum of patients with inflammatory airway disease.

Markers	Ref.	Detection	Study patients	Main findings
MDA	[34]	LC-MS/MS	Asthma, COPD	(i) Increase in stable asthma and COPD (ii) No relationship with LF
	[35]	HPLC	COPD, AECOPD	(i) Increase in stable COPD and AECOPD (ii) Decrease upon recovery from AECOPD (iii) No relationship with LF or sputum differential cell counts
	[36]	HPLC	CF	(i) Increase in stable CF (ii) No relationship with LF or sputum differential cell counts (iii) No association with NE/*α*_1_-PI complex

Hexanal, heptanal, and nonanal	[34]	LC-MS/MS	Asthma, COPD	(i) Increase in hexanal and nonanal in stable COPD (ii) Increase in hexanal in stable asthma

4-HHE, 4-HNE	[34]	LC-MS/MS	Asthma, COPD	No change in stable asthma or COPD

Acrolein	[34]	LC-MS/MS	Asthma, COPD	Increase in stable asthma and COPD^#^

8-Isoprostane	[41]	EIA	Asthma (stable and acute), bronchiectasis	(i) Increase in stable asthma and bronchiectasis (ii) Further increase in acute asthma and decrease upon treatment (iii) Correlation between FEV_1_ (% pred.) and log_10_FGF_2*α*_
	[42]	EIA	HS, COPD	(i) Increase in HS and stable COPD (ii) Relationship with LF, PYI, and sputum neutrophils
	[43]	EIA	HS, COPD	(i) Increase in HS and stable COPD (ii) No effect of aminoguanidine
	[44]	EIA	Asthma, COPD	Increase in COPD but not mild asthma
	[45]	EIA	Asthma	No change in asthma
	[46]	EIA	COPD, AECOPD	(i) Increase in AECOPD compared to stable COPD (ii) Relationship with sputum neutrophils and lymphocytes in stable COPD (iii) No correlations with LF
	[48]	EIA	CF	(i) Increase in acute but not stable CF (ii) No effect of antibiotic treatment

Nitrosothiols	[59]	EIA	COPD	(i) Increase in stable COPD (ii) Positive correlation with GSSG, but negative correlation with GSH in sputum
	[60]	EIA	NAEB, CVA	Higher in NAEB than in CVA

3-NT	[66]	IC	Asthma, COPD	(i) Increase in positive cells in stable asthma and COPD^#^ (ii) Negative correlation with FEV_1_ in COPD
	[67]	IC	HS	Increase in positive cells in HS compared to nonsmokers
	[68]	IC	AECOPD	Increase in positive cells in AECOPD
	[69]	IC	Asthma	(i) Increase in positive cells in refractory asthma compared to well-controlled asthma (ii) Negative correlation with FEV_1_ (% pred)
	[70]	IC	Asthma, COPD	(i) No difference in positive cells between eosinophilic and noneosinophilic asthma (ii) Decrease in positive cells in noneosinophilic asthma compared to COPD
	[71, 72]	HPLC, GC-MS	CF	Increase in stable CF

8-OHdG	[81]	ELISA	COPD, HS, and bronchiectasis	(i) Increase in stable COPD compared to HS and bronchiectasis (ii) Increase in HS compared to nonsmokers
	[82]	ELISA	Asthma, HS	Increase in stable asthma and HS compared to nonsmokers

MDA: malondialdehyde, LC-MS/MS: liquid chromatography-tandem mass spectrometry, COPD: chronic obstructive pulmonary disease, AECOPD: acute exacerbation of chronic obstructive pulmonary disease, HPLC: high-performance liquid chromatography, CF: cystic fibrosis, NE/*α*_1_-PI: neutrophil elastase/*α*_1_-proteinase inhibitor, LF: lung function, 3-NT: 3-nitrotyrosine, EIA: enzyme immunoassay, 4-HHE: 4-hydroxyhexanal, 4-HNE: 4-hydroxynonenal, FEV_1_: forced expiratory volume in one second, HS: healthy smokers, PYI: pack-year index, IC: immunocytostaining, 8-OHdG: 8-hydroxy-2′-deoxyguanosine, GSH: glutathione, GSSG: glutathione disulfide, NAEB: nonasthmatic eosinophil bronchitis, CVA: cough variant asthma, and ELISA: enzyme-linked immunosorbent assays; ^#^markers and/or positive cells not detectable in healthy controls.
